# The Study of Prevalence and Pattern of Thyroid Disorder in Pregnant Women: A Prospective Study

**DOI:** 10.7759/cureus.16457

**Published:** 2021-07-18

**Authors:** Preeti Gupta, Manila Jain, Vandana Verma, Nand K Gupta

**Affiliations:** 1 Department of Physiology, Index Medical College, Hospital and Research Centre, Indore, IND; 2 Obstetrics and Gynecology, Uttar Pradesh University of Medical Sciences, Saifai, Etawah, IND; 3 Anatomy, Uttar Pradesh University of Medical Sciences, Saifai, Etawah, IND

**Keywords:** hyperthyroidism, hypothyroidism, pregnancy, pregnancy-induced thyroid dysfunction, thyroid dysfunction

## Abstract

Background

The most frequent thyroid disorder in pregnancy is maternal hypothyroidism. The geographical variation in the prevalence of hypothyroidism during pregnancy is very wide and ranges from 2.5% to 11%. The prevalence of hypothyroidism is more in Asian countries as compared to western countries. Thus, this study was conducted to find out the prevalence of thyroid disorder in pregnancy at our center.

Methods

The present study was conducted in the Department of Physiology in collaboration with the Department of Obstetrics & Gynecology, Index Medical College, Hospital and Research Center, Indore, MP, India over a period of one and a half years from October 2018 to March 2020. It was a cross-sectional study including 865 pregnant women. The patients' demographic profile was noted in all cases. A detailed history and thorough examination were done in all cases. Serum thyroid-stimulating hormone (TSH), Free T3, and Free T4 (FT3 and FT4) were done along with routine blood investigations as per The Federation of Obstetric and Gynaecological Societies of India-Indian College of Obstetricians and Gynaecologists (FOGSI-ICOG) good clinical practice recommendation.

Results

In this study, the prevalence of thyroid dysfunction was 10.4%. Of these 90 patients with thyroid dysfunction, subclinical and overt hypothyroidism was found in 5.50% and 0.92%, respectively, whereas subclinical and overt hyperthyroidism was observed in 3.12% and 0.81% pregnant females, respectively. A significant association was found between thyroid dysfunction and maternal age, BMI, parity, and education.

Conclusions

The prevalence of thyroid dysfunction was 10.4% in this study. Hypothyroidism was more common than hyperthyroidism and subclinical thyroid disorders were more common than overt thyroid disorders in pregnancy. Therefore, we should include thyroid function tests with other routine investigations during pregnancy to detect thyroid dysfunction.

## Introduction

The most frequent thyroid disorder in pregnancy is maternal hypothyroidism. The geographical variation in the prevalence of hypothyroidism during pregnancy is very wide and ranges from 2.5% to 11% [1]. The prevalence of hypothyroidism is more in Asian countries as compared to western countries. Untreated or inadequately treated and subclinical hypothyroidism all can increase the risk of miscarriage, preeclampsia, anemia, fetal growth restriction, placental abruption, perinatal and neonatal morbidity and mortality, preterm delivery, small head circumference, and low birth weight impaired neuropsychological development [2]. Hypothyroidism consists of two clinical forms: subclinical and overt hypothyroidism. The subclinical hypothyroidism is characterized by an elevated serum thyroid-stimulating hormone (TSH) with normal free thyroxine (FT4) and is observed in 3%-5% of women in pregnancy. Overt hypothyroidism is characterized by an elevated serum TSH and subnormal FT4 is observed in 0.3%-0.5% of women in pregnancy [3,4].

The occurrence of hyperthyroidism is less during pregnancy with the prevalence being 0.1%-0.4%. Overt hyperthyroid­ism is seen in nearly 2% of pregnancy characterized by a reduced TSH and an increased FT3/FT4 while subclinical hyper­thyroidism is seen in 1.7% of pregnancy and is characterized by a suppressed serum TSH and normal FT4 [5]. Physiological changes during pregnancy may mimic hyperthyroidism like fatigue, anxiety, increase in basal metabolism, heart rate, palpitations, heat intolerance, warm and wet skin, hand tremors, and systolic murmur, which causes difficulty in diagnosis [6,7]. Pregnant women suffering from hyperthyroidism have more severe tachycardia and thyromegaly, along with exophthalmia, and lack of weight gain despite having adequate food [1]. Thyroid dysfunction is usually overlooked and ignored in pregnant women because of the non-specific and hypermetabolic state of pregnancy [8]. Thus, this study was conducted to find out the prevalence of thyroid disorders in pregnancy at our center.

## Materials and methods

The present study was conducted in the Department of Physiology in collaboration with the Department of Obstetrics and Gynecology, Index Medical College, Hospital and Research Center, Indore, MP, India over a period of one and a half years from October 2018 to March 2020. It was a cross-sectional study including 865 pregnant women attending clinics in the study period. All antenatal women in their first-trimester pregnancy were included after taking consent except patients with known thyroid disorders, multiple gestations, hypertension, diabetes mellitus, and other medical disorders. The patient’s demographic profile was noted in all cases. After a detailed history and thorough examination, screening for thyroid disorder was done with serum TSH assay along with other routine investigations of pregnancy as per The Federation of Obstetric and Gynaecological Societies of India-Indian College of Obstetricians and Gynaecologists (FOGSI-ICOG) good clinical practice recommendation. Those with abnormal TSH were subjected to FT4, FT3, and anti-thyroid peroxidase antibody assay. Women diagnosed with abnormal thyroid functions were referred to the endocrinology department for the treatment of thyroid dysfunction. Hypothyroid patients (subclinical and overt variety) were treated with levothyroxine while hyperthyroidism was treated with propylthiouracil. Repeat thyroid profiles were done at 4-6 weeks intervals and treatment was adjusted to keep the serum TSH levels within normal limits.

The reference range used in the study was based on the guidelines of the American Thyroid Association (ATA) 2017 [9]. According to which normal levels of TSH during 1st, 2nd, and 3rd trimester of pregnancy are 0.1-2.5 mIU/L, 0.2-3.0 mIU/L, and 0.3-3.0 mIU/L, respectively, and normal levels of FT4 and FT3 during pregnancy are 0.7-1.8 pg/mL and 1.7-4.2 pg/mL, respectively. Depending on the normal values, patients were classified into subclinical hypothyroidism (high serum TSH with normal FT4, FT3 level), overt hypothyroidism (high serum TSH with FT4, FT3 less than normal), subclinical hyperthyroidism (low serum TSH with normal FT3, FT4), and overt hyperthyroidism (low serum TSH with FT4 and FT3 more than normal range). The data were analyzed using the IBM SPSS version 23 (Armonk, NY). Discrete (categorical) groups, compared by Chi-squared test (χ2) to correlate demographic distribution in groups. P-value less than 0.05 (p < 0.05) was considered statistically significant.

## Results

In our study, 90 patients out of 865 pregnant women have a thyroid disorder. The prevalence of thyroid dysfunction in our study was 10.40% in which the prevalence of hypothyroidism and hyperthyroidism was 6.47% and 3.93%, respectively. Of these 90 patients with thyroid dysfunction, hypothyroidism was diagnosed in 56 (62.2%) whereas hyperthyroidism was in 34 (37.8%) patients. Subclinical hypothyroidism and overt hypothyroidism were observed in 48 (53.3%) and 8 (8.9%) patients, respectively, while subclinical and overt hyperthyroidism was observed in 27 (30%) and 7 (7.78%) women, respectively.

Patients with subclinical hypothyroidism, overt hypothyroidism, overt hyperthyroidism were more common in the age group of more than 30 years while patients with subclinical hyperthyroidism were more common in the age group of less than 30 years and significant distribution was observed between thyroid disorders with age (Table 1).

**Table 1:** Distribution of thyroid disorders with age group. * Chi-squared test/Fisher's exact test value 27.036, degree of freedom = 9.

Thyroid disorders		Frequency (n = 90)	Age group (years)				P-value*
			<25	26-30	31-35	35-40	
Hypothyroidism	Subclinical	48	7 (14.6%)	13 (27.1%)	14 (29.2%)	14 (29.2%)	<0.001
	Overt	8	0 (0.0%)	0 (0.0%)	7 (87.5%)	1 (12.5%)	
Hyperthyroidism	Subclinical	27	11 (40.7%)	7 (25.9%)	6 (22.2%)	3 (11.1%)	
	Overt	7	0 (0.0%)	0 (0.0%)	5 (71.4%)	2 (28.6%)	

There is a causal relationship between BMI and thyroid dysfunction. All types of thyroid dysfunction were more common in overweight and obese patients, and thyroid disorder with BMI distribution was observed significantly (Table 2).

**Table 2:** Distribution of thyroid disorder with BMI (kg/m2). * Chi-squared test/Fisher's exact test value 23.438, degree of freedom = 6.

Thyroid disorders		Frequency (n = 90)	BMI (kg/m^2^)			P-value*
			<25	26-30	>30	
Hypothyroidism	Subclinical	48	0 (0.0%)	18 (37.5%)	30 (62.5%)	<0.001
	Overt	8	0 (0.0%)	0 (0.0%)	8 (100.0%)	
Hyperthyroidism	Subclinical	27	6 (22.2%)	6 (22.2%)	15 (55.6%)	
	Overt	7	0 (0.0%)	0 (0.0%)	7 (100.0%)	

The majority of the subclinical and overt hypothyroidism patients were multipara, while subclinical and overt hyperthyroidism was more common in the primipara women group and association was significant (Table 3).

**Table 3:** Distribution of thyroid disorder with parity. * Chi-squared test/Fisher's exact test value 10.071, degree of freedom = 3.

Thyroid disorders		Frequency (n = 90)	Parity		P-value*
			Single (primipara)	Multiple (multipara)	
Hypothyroidism	Subclinical	48	18 (37.5%)	30 (62.2%)	<0.018
	Overt	8	3 (37.5%)	5 (62.5%)	
Hyperthyroidism	Subclinical	27	18 (66.7%)	9 (33.3%)	
	Overt	7	6 (85.7%)	1 (14.3%)	

Maximum cases of subclinical hypothyroidism and overt hyperthyroidism patients were found in the educated group (Primary to Graduate) while subclinical hyperthyroidism and overt hypothyroidism patients were found in the Illiterate group and the distribution of thyroid disorder with education was significant (Table 4).

**Table 4:** Distribution of thyroid disorders with education status. * Chi-squared test/Fisher's exact test value 35.029, degree of freedom = 6.

Thyroid disorders		Frequency (n = 90)	Education			P-value*
			Illiterate	Primary and Middle	Secondary and Graduate	
Hypothyroidism	Subclinical	48	13 (27.1%)	26 (54.2%)	9 (18.8%)	<0.001
	Overt	8	6 (75.0%)	0 (0.0%)	2 (25.0%)	
Hyperthyroidism	Subclinical	27	21 (77.8%)	1 (3.7%)	5 (18.5%)	
	Overt	7	3 (42.9%)	0 (0.0%)	4 (57.1%)	

## Discussion

In our study, the prevalence of thyroid dysfunction was found to be 10.40% and this was comparable by various other Indian and foreign studies (Table 5) [4,10,11-13].

**Table 5:** Prevalence of thyroid dysfunction.

Studies	Prevalence
Mulik J et al. [10]	12.15%
Sahu MT et al. [11]	12.7%
Azenabor A et al. [12]	11.1%
Singh A and Reddy MJ [4]	8.25%
Chunchaiah S et al. [13]	11.25%
Present study	10.40%

The mean age in this study was 31.30 ± 4.92 years, which was similar to various other studies (Table 6) [12,14,15].

**Table 6:** Comparison of age of participants in various previous studies.

Studies	Mean age
Azenabor A et al. [12]	29.82 ± 4.39 years
Abdulslam K et al. [14]	27.7 ± 7.8 years
Irinyenikan TA et al. [15]	30.4 ± 4.62 years
Present study	31.30 ± 4.92 years

In this study, the prevalence of subclinical hypothyroidism and overt hypothyroidism was 5.50% and 0.92% respectively, while subclinical hyperthyroidism and overt hyperthyroidism were 3.12% and 0.81% respectively. Thus hypothyroidism was found more common in pregnant women than hyperthyroidism in our study, which was similar to the studies conducted by Sahu MT et al., Abdulslam K et al., Gayathri R et al., and Nazarpour S et al., and on different sample sizes (Table 7) [11,14,16,17].

**Table 7:** Pattern of thyroid disorders in various studies.

Studies	Sample size	Hypothyroidism		Hyperthyroidism	
		Subclinical	Overt	Subclinical	Overt
Sahu MT et al. [11]	633	6.5%	4.6%	-	-
Gayathri R et al. [16]	500	6.47%	-	-	-
Nazarpour S et al. [17]	Meta analysis of 512 article	-	-	1.7%	0.2%
Abdulslam K et al. [14]	165	4.8%	1.6%	1.6%	-
Present study	865	5.50%	0.92%	3.12%	0.81%

The distribution of various types of thyroid dysfunction with respect to age was found to be statistically significant (p-value < 0.001) which was similar to a study done by Ajmani SN et al. who reported an increased occurrence of thyroid dysfunction with advanced maternal age [18]. Abd Elwahid Suliman AA et al. also reported that older women were more likely to have hypothyroidism than younger [19].

In the study of Dulek H et al., correlation analysis was performed for TSH elevation using some parameters. Correlation analyses were performed between TSH values and parameters such as maternal age (r = 0.085, p = 0.04), anti-TPO (r = 0.347, p = 0.09), and birth weight (r = -0.07, p = 0.873). An increase in TSH correlated positively with maternal age [20].

Contrary to this in the study of Diéguez M et al., they observed no difference in TSH and FT4 mean values between women <30 years and ≥30 years. Mean TSH in women <30 years was 2-21 mIU/l, and in women ≥30 years, it was 213 mIU/l, (T: 0.98, mean difference: 0.08, P: 0.32). Mean FT4 was 14.9 pmol/l and 15.1 pmol/l in the two age groups, respectively, (T: 1.28, mean difference: 0.009, P: 0.19) [21]. Potlukova et al. and Ezzeddine D et al. also conducted studies on thyroid disorder among antenatal women and found no significant association between age and thyroid dysfunction [22,23].

In our study, we observed a statistically significant distribution (P-value < 0.001) of BMI with the women suffering from thyroid dysfunction which was contrary to Pillai NS and Bennett J study in which they observed an increased risk of hypothyroidism in pregnancy as the BMI increases and the p-value was statistically significant (p = 0.015) [24]. A study by Abdulslam K et al. and Ajmani SN et al. also showed an increase in the incidence of thyroid dysfunction with increasing BMI [14,18]. This shows that BMI plays a significant role in the prevalence of thyroid dysfunction, as the BMI increases, the prevalence of thyroid dysfunction also increases significantly.

In this study, the majority of the subclinical and overt hypothyroidism patients was found in multiple parity women while subclinical and overt hyperthyroidism was more common in single parity women and association was statistically significant (p < 0.05) which was similar to Abd Elwahid Suliman AA et al. study in which they reported a significant association between parity and overt hypothyroidism [19]. While the study of Prasad DR et al. showed no statistically significant difference (p > 0.05) between parity and hypothyroidism and Nirmala C et al. also found that maternal outcome of hypothyroidism in pregnancy of South Indian females have no statistical difference with respect to parity in different groups [25,26].

In our study, the majority of the subclinical hypothyroidism and overt hyperthyroidism patients was found in the Educated group (Primary to Graduate) while subclinical hyperthyroidism and overt hypothyroidism patients were found in the Illiterate group and the association was significant (p < 0.05) which was similar to the study of Abd Elwahid Suliman AA et al. [19]. However, in the studies of Behrooz HG et al. and Manju VK and Sathiamma PK, they reported an insignificant association (p > 0.05) between educational status and thyroid disorders [27,28].

## Conclusions

In our study, the prevalence of thyroid dysfunction in pregnant women was found to be 10.40% and the prevalence of subclinical hypothyroidism and overt hypothyroidism was found to be 5.50% and 0.92%, respectively, while subclinical hyperthyroidism and overt hyperthyroidism was 3.12% and 0.81%, respectively. In our study, we conclude that subclinical hypothyroidism is more common than hyperthyroidism in pregnant women. Therefore, we should include thyroid function tests with other routine investigations during pregnancy to detect thyroid dysfunction.

## References

[1] Altomare M, La Vignera S, Asero P (2013). High prevalence of thyroid dysfunction in pregnant women. J Endocrinol Invest.

[2] Unnikrishnan AG, Kalra S, Sahay RK, Bantwal G, John M, Tewari N (2013). Prevalence of hypothyroidism in adults: an epidemiological study in eight cities of India. Indian J Endocrinol Metab.

[3] Reid SM, Middleton P, Cossich MC, Crowther CA (2010). Interventions for clinical and subclinical hypothyroidism in pregnancy. Cochrane Database Syst Rev.

[4] Singh A, Reddy MJ (2015). Prevalence of thyroid dysfunction in pregnancy and its implications. Int J Med Sci Public Health.

[5] Brent GA (2007). Diagnosing thyroid dysfunction in pregnant women: is case finding enough?. J Clin Endocrinol Metab.

[6] Casey BM, Leveno KJ (2006). Thyroid disease in pregnancy. Obstet Gynecol.

[7] Alamdari S, Azizi F, Delshad H, Sarvghadi F, Amouzegar A, Mehran L (2013). Management of hyperthyroidism in pregnancy: comparison of recommendations of American Thyroid Association and Endocrine society. J Thyroid Res.

[8] Jani RS, Munshi DS, Jani SK, Munshi SP, Solanki SB, Pandya VM (2014). Prevalance and fetomaternal outcome of thyroid disorder in pregnancy. Int J Med Sci Public Health.

[9] Alexander EK, Pearce EN, Brent GA (2017). 2017 guidelines of the American Thyroid Association for the diagnosis and management of thyroid disease during pregnancy and the postpartum. Thyroid.

[10] Mulik J, Pratapan P, Agrawal N (2017). Study of thyroid disorders in pregnancy & its effect on maternal & perinatal outcomes at a tertiary care centre. PIJR.

[11] Sahu MT, Das V, Mittal S, Agarwal A, Sahu M (2010). Overt and subclinical thyroid dysfunction among Indian pregnant women and its effect on maternal and fetal outcome. Arch Gynecol Obstet.

[12] Azenabor A, Omumene JP, Ekun AO (2016). Pattern and prevalence of thyroid dysfunction in Nigerian pregnant females. J Adv Med Med Res.

[13] Chunchaiah S, Prasad N, Murali B M, Rupakala BM, Rangaiah N (2016). A prospective observational study of thyroid dysfunctions during pregnancy in a tertiary care hospital. Int J Reprod Contracept Obstet Gynecol.

[14] Abdulslam K, Yahaya IA (2015). Prevalence of thyroid dysfunction in gestational hypertensive Nigerians. Sub-Saharan Afr J Med.

[15] Irinyenikan TA, Arowojolu A, Olayemi O (2014). Comparative study of serum lipid levels in normotensive and pre-eclamptic Nigerian women. Int J Med Biomed Res.

[16] Gayathri R, Lavanya S, Raghavan K (2009). Subclinical hypothyroidism and autoimmune thyroiditis in pregnancy--a study in south Indian subjects. J Assoc Physicians India.

[17] Nazarpour S, Ramezani Tehrani F, Simbar M, Azizi F (2015). Thyroid dysfunction and pregnancy outcomes. Iran J Reprod Med.

[18] Ajmani SN, Aggarwal D, Bhatia P, Sharma M, Sarabhai V, Paul M (2014). Prevalence of overt and subclinical thyroid dysfunction among pregnant women and its effect on maternal and fetal outcome. J Obstet Gynaecol India.

[19] Abd Elwahid Suliman AA, Albasha MA, Ibrahim HS, Handady SM, Alawad AM (2018). Rate, pattern and risk factors of hypothyroidism among Sudanese pregnant women. Austin J Obstet Gynecol.

[20] Dulek H, Vural F, Aka N, Zengin S (2019). The prevalence of thyroid dysfunction and its relationship with perinatal outcomes in pregnant women in the third trimester. North Clin Istanb.

[21] Diéguez M, Herrero A, Avello N, Suárez P, Delgado E, Menéndez E (2016). Prevalence of thyroid dysfunction in women in early pregnancy: does it increase with maternal age?. Clin Endocrinol (Oxf).

[22] Potlukova E, Potluka O, Jiskra J, Limanova Z, Telicka Z, Bartakova J, Springer D (2012). Is age a risk factor for hypothyroidism in pregnancy? An analysis of 5223 pregnant women. J Clin Endocrinol Metab.

[23] Ezzeddine D, Ezzeddine D, Hamadi C, Abbas HA, Nassar A, Abiad M, Ghazeeri G (2017). Prevalence and correlation of hypothyroidism with pregnancy outcomes among Lebanese women. J Endocr Soc.

[24] Pillai NS, Bennett J (2018). Prevalence of hypothyroidism amongst pregnant women: a study done in rural set up. Int J Reprod Contracept Obstet Gynecol.

[25] Prasad DR, Nair NV, Deepika K (2016). A descriptive study of the prevalence of hypothyroidism among antenatal women and foetal outcome in treated hypothyroid women. Int J Reprod Contracept Obstet Gynecol.

[26] Nirmala C, Jayakumari C, Rajasekharan C, Nandini VR (2014). Maternal outcome of hypothyroidism in pregnancy- a south Indian perspect. Am J Clin Med Res.

[27] Behrooz HG, Tohidi M, Mehrabi Y, Behrooz EG, Tehranidoost M, Azizi F (2011). Subclinical hypothyroidism in pregnancy: intellectual development of offspring. Thyroid.

[28] Manju VK, Sathiamma PK (2017). Maternal outcome in thyroid dysfunction. Int J Reprod Contracept Obstet Gynecol.

